# *Citrus aurantium* Ameliorates Cisplatin-Induced Nephrotoxicity

**DOI:** 10.1155/2019/3960908

**Published:** 2019-12-06

**Authors:** Rui Wang, Waseem Hassan, Fiaz ud Din Ahmad, Qaiser Jabeen, Hammad Ahmed, Omer Iqbal

**Affiliations:** ^1^Department of Nephrology, Zhenzhou Central Hospital, Affiliated to Zhengzhou University, Zhengzhou City, Henan Province 450000, China; ^2^Department of Pharmacy, COMSATS University Islamabad, Lahore Campus, Lahore 54000, Pakistan; ^3^Department of Pharmacy, The Islamia University of Bahawalpur, Bahawalpur 63100, Pakistan; ^4^Faculty of Pharmacy, The University of Lahore, Lahore 54000, Pakistan

## Abstract

We aimed to study the effects of *Citrus aurantium* (*C. aurantium*) on renal functions in cisplatin-induced nephrotoxicity in rats. The study involved male Wistar rats weighing 250–300 g that were fed and kept under standard conditions. Rats were randomly divided into control, cisplatin administered, *C. aurantium* 200 mg/kg, and *C. aurantium* 400 mg/kg groups. Cisplatin was administered at 5 mg/kg i.p. once at the start of study to induce nephrotoxicity. Blood and urine samples were obtained at alternative days for analysis. The body weight and urine output were monitored at regular intervals. Plasma and urinary sodium, potassium, and creatinine levels were measured at the end of study duration. Absolute excretion of sodium and potassium; sodium to potassium ratio; kidney weights; fractional excretion of sodium and potassium; and absolute creatinine clearance were determined to analyze the effects of *C. aurantium*. Histopathological changes of kidney tissues were studied to determine relevant effects. The results indicate that cisplatin lowered the total body weights while raising the urinary output and kidney weights, reversed by *C. aurantium* both dose and time dependently. Similarly, *C.* aurantium markedly normalized plasma, urinary sodium, potassium, and creatinine levels. Cisplatin-induced absolute sodium clearance, absolute potassium clearance, absolute creatinine clearance, sodium to potassium ratio, and fractional excretion of sodium and potassium were significantly reversed by *C.* aurantium. Histopathological analysis showed notable improvement in *C.* aurantium administered groups as compared to cisplatin-induced group. Study suggests that *C. aurantium* possesses excellent nephroprotective effects against cisplatin-induced toxicity.

## 1. Introduction

Cis-diamminedichloroplatinum (II) is inorganic platinum which is widely used as a potent chemotherapeutic drug [1]. It is an alkylating agent that reacts with DNA to form interstrand cross-links and intrastrand bifunctional N-7 DNA adducts at d(GpG) and d(ApG) [2]. Thus far it has shown its effectiveness in testicular cancer, ovarian cancer, cervical cancer, breast cancer, bladder cancer, head and neck cancer, esophageal cancer, lung cancer, germ cell tumors, lymphomas, and sarcomas, brain tumors, and neuroblastoma [3]. Despite its large-scale effectiveness in malignancies, cisplatin-induced (CIN) toxicities remain a single-most limiting factor in its use in cancer therapies. It causes toxicities of gastrointestinal [4], renal [5], neurological [6], hepatic [7], and haematological systems [8], even when administered at normal doses. Despite a wide range of side effects, nephrotoxicity remains a prominent reason for its discontinuation in malignancies as its prevalence touches one-third of treated patients with dose-limiting effects [9, 10]. For instance, moderate to severe nephrotoxicity was observed in 25%–33% of patients at the dose of 50–75 mg/m^2^ [11]. Even at much lower doses of 20 mg/m^2^ IV for five days, most of the patients (50%–70%) suffered moderate to severe nephrotoxicity [12]. Expectedly, incidence of nephrotoxicity increased as cisplatin dose was raised [13]. Clinically, it appears nearly after 10 days of treatment. Due to its nephrotoxic potential, it is generally restrained to patients having creatinine clearance (CrCl) > 60 Ml/min. There are other risk factors like co-treatment with other nephrotoxins such as aminoglycosides, NSAIDs, and streptozocin [14] that adds to the already raised chances of cisplatin-induced nephrotoxicity.

A numbers of mechanisms are reported to be responsible for CIN, among which overproduction of reactive oxygen species (ROS) and vasoconstriction in the renal microvasculature are reported accountable for the cisplatin-induced renal tubular injury. Furthermore, S3 segment of the proximal tubule on the external band of the outer medulla is selectively injured by cisplatin. Beyond that, generation of reactive nitrogen species (NOS) have also been reported in CIN which incite alterations in the operation and structure of lipid peroxidation and chemical cleavage of DNA and proteins [15].

There are fewer therapies that can be offered to tolerate the cisplatin-induced toxicity. The standard approach is to reduce the doses of cisplatin in combination with IV hydration before and after cisplatin administration which significantly decreases cisplatin half-life, urinary cisplatin concentration, and proximal tubule transit time [16–18]. Slow infusion rate and concomitant administration of mannitol can also reduce the severity of cisplatin-induced toxicity [19]. Moreover, antioxidant therapies are indicated in the literature to reduce the intensity of CIN and make it more tolerable for cancer patients. One such robust study presented by Nematbakhsh et al. demonstrated that selenium and vitamin E which are antioxidants are effective in curtailing oxidative toxicity of cisplatin [20]. There are other evidences which suggest that ROS and mitochondrial damage is at least partially responsible for CIN [21, 22].

*C. aurantium* is a traditional plant used for centuries to treat indigestion, diarrhea dysentery, constipation, and dry cough [23, 24]. It is mentioned in South American folk medicine as therapeutic agent for insomnia, anxiety, and epilepsy [25]. Research has also highlighted their pharmacological effects as an antioxidant, cardioprotective, antiproliferative, anticancer, and hypolipidemic agent [26–29]. Due to its antioxidative and anticancer potentials, it becomes a natural therapeutic agent for CIN. Hence, this study is designed to evaluate the pharmacological potential of *C. aurantium* in CIN.

## 2. Materials and Methods

### 2.1. Animals

Wistar albino rats, weighing 250–300 g, were kept in the experimental research laboratory, in the Islamia University of Bahawalpur, under 12 h light/dark cycles. The standard humidity (45–65%) and temperature (22–24°C) conditions were maintained. All the mice were provided with water and standard pellet diet ad libitum. Approvals of all the experimental protocols were taken from the Ethical Review Committee, Islamia University of Bahawalpur.

### 2.2. Materials

Chemicals of analytical grade used for research purpose included cisplatin (Pfizer Laboratory LTD), 2, 2-diphenyl, 1-picrylhydrazyl (DPPH), formalin, ketamine (Indus pharma Lahore), xylazine (prix pharmaceutical, Lahore), ether, aqueous ethanol, picric acid, sodium hydroxide, and trichloroacetic acid which all are of analytical grade.

### 2.3. Preparation of Extract

The peels of *citrus aurantium* were separated, dried under shade for 15 days, and powdered in a blender. It was then grinded by an electric grinder and soaked in a mixture of 60% ethanol for three days with shaking and agitation occasionally. Obtained residue is then evaporated under reduced pressure and at temperature of 30–40°C by using a rotary evaporator. Semisolid residue obtained was then kept in the refrigerator till further analysis.

### 2.4. Experimental Protocol

Rats were randomly divided into four groups containing six animals each. Group-I was kept untreated and received normal saline via oral route for 21 days. Group-II was considered intoxicated group administered only with cisplatin at 5 mg/kg i.p. on day 1. Group-III and Group-IV were treated with *Citrus Aurantium* extract at the oral doses of 200 mg/kg and 400 mg/kg once daily, respectively, for 21 days in the presence of cisplatin-induced toxicity.

### 2.5. Blood Samples and Metabolic Data Collection

For the measurement of sodium and potassium levels (metabolic data) in all the experimental groups, urine was sampled on 0, 7^th^, 14^th^, and 21^st^ days of the study. Rats were kept in metabolic cages for 24 hours with free access to tap water. Water intake and urine output were measured regularly. For the estimation of creatinine, sodium, and potassium levels, samples were kept at −30°C. The flame photometer (Sherwood model 410, UK) was used to measure the levels of sodium and potassium in plasma and urine samples. For the measurement of sodium and potassium in plasma, samples were diluted as 1 : 200. Identical dilution was made to estimate potassium levels in urine but for the sodium level, the 1 : 1000 dilution was used. Creatinine was measured using the spectrophotometer method at the wave length of 520 nm.

### 2.6. Histopathology

A section of the kidney was fixed in 10% V/V neutral-buffered formalin and then processed for dehydration by passing them through pools of ethanol having different concentrations. Then, paraffin blocks were prepared and 5 *μ*m thick sections were cut for staining with hematoxylin and eosin (H&E).

### 2.7. Statistical Analysis

The values are shown as mean ± SEM of 6 animals in each group. The results are evaluated by using one-way ANOVA followed by Bonferroni post hoc test and then compared with the normal control group. The results are mainly considered significant (^*∗*^) if *p* < 0.05, more significant (^*∗∗*^) if *p* < 0.01, and highly significant (^*∗∗∗*^) if *p* < 0.001.

## 3. Results

### 3.1. *C*. *aurantium* Effects on Physical Features of Cisplatin-Administered Rats

Cisplatin notably reduced the body weights and pattern continued till the end of study duration. It is worth noticing that the control group followed the natural trend as body weights kept growing till the end of study. *C. aurantium* at its highest dose was able to significantly curtail the weight loss (Table 1). Similarly cisplatin increased the daily urinary output, while *C. aurantium* at both doses controlled the urinary output (Table 2). It was noticed that kidney weight was incomparably elevated in the animals associated with the cisplatin group when contrasted with normal control. Co-administrating *C. aurantium* extract at different doses with cisplatin caused slight decrease in kidney weight (Table 3).

### 3.2. *C. aurantium* Normalizes the Plasma and Urinary Sodium and Potassium Levels of Cisplatin-Administered Rats

Cisplatin-induced rats reduced the plasma sodium levels down to 103 mEq/L as compared to the control group which remained at 139 mEq/L. Comparatively, *C. aurantium* raised the cisplatin-induced suppression of plasma sodium at increasing doses. Plasma potassium also followed similar pattern and cisplatin-inhibited levels were brought to normal by *C. aurantium* (Table 4). On the contrary, urinary sodium and potassium levels were notably increased, which were expectedly brought down by *C*. *aurantium* (Table 5).

### 3.3. *C. aurantium* Regulates Plasma and Urinary Creatinine Levels

**Figure 1:**
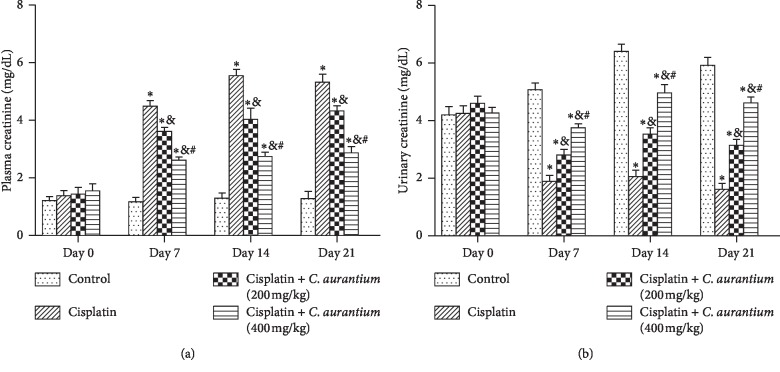
Effects of *C. aurantium* on plasma (a) and urinary (b) creatinine levels. The values are mean ± SEM (*n* = 6). Statistical analysis was done through one-way analysis of variance (ANOVA) trailed by Bonferroni post hoc test for all groups in respective days. The results are considered significant (^*∗*^) if *p* < 0.005. ^*∗*^*p* < 0.05 vs. normal control, ^&^*p* < 0.05 vs. cisplatin, and ^#^*p* < 0.05 vs. cisplatin + *C. aurantium* on corresponding days.

The cisplatin-induced animal group showed raised plasma creatinine levels as compared to control. However *C. aurantium* at both of its studied doses remarkably reduced the plasma creatinine levels (Figure 1). On the contrary, cisplatin-induced urinary creatinine was increased by *C. aurantium* (Figure 1). It may be plausible to comment that increased excretion of creatinine leads to lower plasma levels of creatinine.

### 3.4. *C. aurantium* Reduces Absolute Sodium Excretion (U_Na_V)

**Figure 2:**
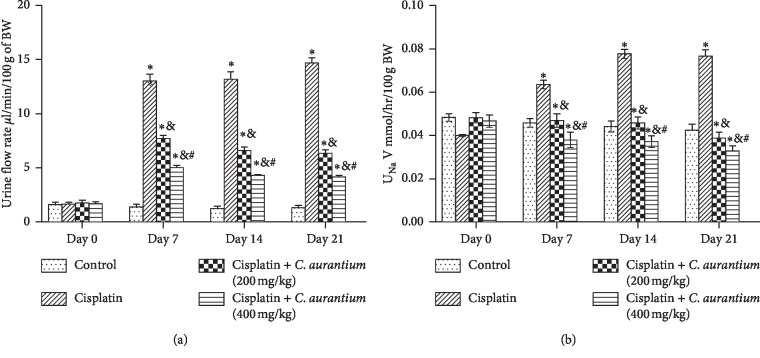
Demonstration of urine flow rate (a); absolute sodium excretion (U_Na_V) (b). The values are mean ± SEM (*n* = 6). Statistical analysis was done through one-way analysis of variance (ANOVA) followed by Bonferroni post hoc test for all groups in respective days. The results are considered significant (^*∗*^) if *p* < 0.005. ^*∗*^*p* < 0.05 vs. normal control, ^&^*p* < 0.05 vs. cisplatin, and ^#^*p* < 0.05 vs. cisplatin + *C. aurantium* (400 mg/kg) on respective days.

*C. aurantium* significantly reduces absolute sodium excretion both dose and time dependently. Maximum sodium excretion is recorded at 21^st^ day which *C. aurantium* was able to reverse at both 200 mg/kg and 400 mg/kg doses (Figure 2(b)). Correspondingly, urinary flow rate was also reduced following a similar pattern. Effects of *C. aurantium* were most pronounced at 400 mg/kg at 21^st^ day (Figure 2(a)).

### 3.5. Effects of *C. aurantium* on Absolute Creatinine and Absolute Potassium Clearance

*C. aurantium* notably restored the cisplatin-induced lower absolute creatinine clearance. The effects at the dose of 400 mg/kg were more pronounced after 21^st^ day as compared to other dose groups, signifying both dose and time dependent effects (Table 6). Similarly, absolute potassium excretion increased by cisplatin, which was brought to almost normal levels by *C. aurantium* at 400 mg/kg (Table 7).

### 3.6. Effects of *C. aurantium* on Urinary Na^2+^/K^+^ Ratio and Fractional Excretion of Sodium and Potassium

The urinary Na^2+^/K^+^ ratio was most notably enhanced by cisplatin as compared to control and treatment groups. *C. aurantium* at 200 mg/kg and 400 mg/kg both dose and time dependently reversed the effects of cisplatin. After 21^st^ day of treatment, the Na/K ratio was determined at 20 for cisplatin group as compared to 6 for *C. aurantium* at 400 mg/kg (Table 8). Similarly, fractional excretion of sodium and potassium was also increased by cisplatin to 26 and 0.69, respectively. *C. aurantium* brought fractional excretion of sodium and potassium back to 13 and 0.35, respectively (Table 9).

### 3.7. Histopathological Analysis of Kidney

**Figure 3:**
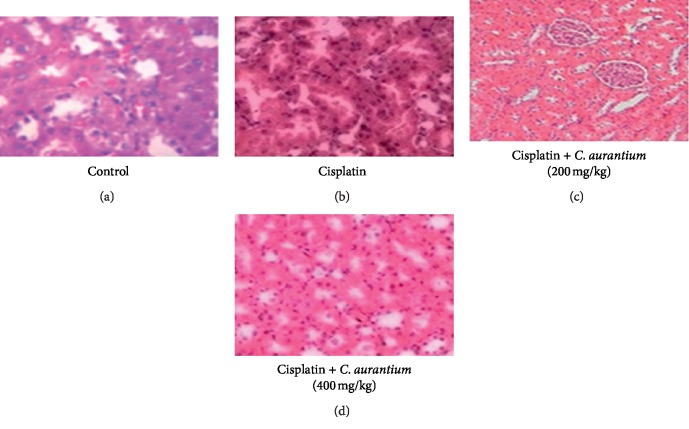
Histopathological sections of the rat kidney. Control (a); cisplatin (b); cisplatin + *C. aurantium* + 200 mg/kg (c); cisplatin + *C. aurantium* + 400 mg/kg (d).

To observe impact of disparate doses of *C. aurantium*, a kidney from every animal was anatomized out for histopathological investigation. The control group showed the intact bowman's capsule, proximal and distal convoluted tubule. No capillary congestion, hemorrhage, and interstitial damage are seen in this group. The cisplatin group indicated extreme tubular and glomerular degeneration alongside putrefaction when contrasted to the control group. While the treatment groups at different doses (200 mg/kg and 400 mg/kg) of CA extract demonstrated checked capacity to keep the cisplatin prompted epithelial damage. 400 mg/kg group showed many glomeruli that are intact with bowman's capsule, and less interstitial damage was seen in proximal and distal convoluted tubules (Figure 3).

## 4. Discussion

The renal system is a routine victim of xenobiotic due to its ability to remove concentrated toxins. The load on the kidney that leads to serious complications is increased by the development of nephrotoxicity [30]. Cisplatin is a widely used inorganic platinum-based potent chemotherapeutic drug which is a great success in the war of cancer [3]. Cisplatin is now being used in the treatment of solid organ malignancies that include the lung, ovarian, testicular, bladder, colorectal, and head and neck cancers [31]. Nephrotoxicity was reported in the earlier clinical trials undergoing cisplatin chemotherapy [32]. Nephrotoxicity is initiated by changes in renal hemodynamics followed by acute, mainly proximal tubular impairment. After 72 hours of treatment with cisplatin, distal and proximal tubular reabsorptive capacities are compromised. Five-day clinical treatment with 20 mg cisplatin/m^2^ per day caused notable reduction in decrease in 51Cr-EDTA clearance. At higher doses of 40 mg/m^2^ day, GFR is severely compromised which remained blunted for up to 2 years even after termination of cisplatin [32]. High-dose administration of cisplatin has showed tendency to cause proteinuria [33]. Proteinuria chiefly belonging to tubular and glomerular origin and tend to occur between treatment cycles. The processes by which cisplatin causes nephrotoxicity are complex and are mediated by various cellular processes that includes electrolyte imbalance and wasting [34], abnormal creatinine clearance [35], oxidative stress [36], apoptosis [37], and inflammation [38].

The medical history has proved that indigenous plants are natural barriers against ailments and serves as a healing reservoir. *C. aurantium* is a natural herb indigenous to Southeast Asia, Bahamas, United States, and Spain. It is consumed as a fruit and has been linked with treatment of various diseases as described in Introduction. The study aims to evaluate the pharmacological potential of *C. aurantium* in cisplatin-induced nephrotoxicity.

Nephrotoxicity was observed by several renal function parameters which was also a contributing factor in mortality rate. Weight loss was observed due to gastrointestinal toxicity and by reduced ingestion of food. Progressive weight loss of animals in the cisplatin-treated group has been closely linked to poor feed, increased catabolism, physiological imbalance, or mental stress [39]. As per previous results, our study also showed weight loss in cisplatin-induced group, which was brought back by *C. aurantium* both dose and time dependently. It is suspected that gastrointestinal damage and increased ingestion of food are responsible for *C. aurantium* antianorexic effects. Similarly, the increase in kidney weight was observed in groups treated with cisplatin correlated with edema or inflammation due to cisplatin-induced tubular necrosis [40]. The weight reduction of the kidney was observed significantly in animals treated with *C. aurantium* which might be due to its anti-inflammatory action [41]. Furthermore, it was observed that the urine volume is increased in animals treated with cisplatin which is related with cisplatin-induced nonoligouric acute renal failure. Cisplatin induces increase in urine output due to reduction in the gene expression of aquaporins and their density in the proximal tubule. The decrease in urine output was observed in groups that received *C. aurantium* as it was also reported previously that increase in urine volume was seen in rats having nephrotoxicity caused by cisplatin [42].

CIN causes debilitation of cell membrane pumps such as sodium potassium pumps which results in reduced reabsorption of sodium eventually raising its concentration in urine [43]. The transport system abnormality may result in hypernatremia and hyperkalemia. In this study, hypernatriuria and hyperkaliuria were presented in cisplatin-treated groups. Administration of *C. aurantium* along with cisplatin causes reduction in levels of sodium and potassium near to normal values as compared to the intoxicated group. Similarly, there was a decrease in sodium and potassium levels in plasma in groups treated with cisplatin as compared to the control group, while *C. aurantium* dose groups brought plasma, sodium, and potassium levels near to normal values.

The study additionally showed the increase in plasma creatinine level and lowered creatinine clearance in cisplatin-treated groups as compared to the control group. This outcome evidently shows the reduction in ability of kidney to filter waste products or to conserve cations abundantly. The alteration in electrolytes and decrease in activity of Na–K ATPase are associated with cisplatin nephrotoxicity which are demonstrated by hyponatremia and hypokalaemia [44]. Expectedly *C. aurantium* reversed the cisplatin-induced changes in creatinine level and creatinine clearance. Our results corresponds with the earlier findings showing lower creatinine clearance and higher creatinine levels in plasma when induced with cisplatin [45].

It was also observed that renal markers such as absolute excretion of sodium and potassium, fractional excretion of sodium cisplatin and potassium, urine to sodium potassium ratio, and absolute creatinine clearance values showed notable disturbance in cisplatin-induced group. *C. aurantium* reversed the values to near normalcy as it is evident in results section. Our results are in line with the previous findings that showed the disturbances in abovementioned parameters [46, 47].

Nephrotoxicity in experimental animals can be affirmed by assessing pathological symptoms such as, for example, tubular degeneration, putrefaction, intertubular drain, desquamation, presence of hyaline casts in tubules, and blockage and swelling in glomerulus. In our study, treatment with cisplatin caused extreme tubular and glomerular degeneration alongside putrefaction when contrasted to the control group. These alterations were reduced in groups that are treated with *C. aurantium*, thus showing its protective effect. It was observed that improvement of cisplatin-incited renal damage was more notable in rats treated with 400 mg/kg of *C. aurantium*. This study leaves a scientific gap for the isolation of *C.* aurantium components and its further determination of mechanism in CIN.

## 5. Conclusion

It may be concluded that *C. aurantium* has the ability to counter the nephrotoxic potential of cisplatin at the experimental dose of 5 mg/kg i.p. We recommend further studies to ascertain the exact mechanism of action as well as to identify the potential active constituent exhibiting the nephroprotective potential.

## Figures and Tables

**Table 1:** Effect of crude extract of *C. aurantium* on body weight in rats treated with cisplatin.

Days of observation				
Groups	0^th^ day	7^th^ day	14^th^ day	21^st^ day
Control	241 ± 7.5	261 ± 8.0	328 ± 8.7	310 ± 8.2
Cisplatin	245 ± 8.0	187 ± 9.2^*∗∗∗*^	198 ± 11^*∗∗∗*^	163 ± 8.4^*∗∗∗*^
Cisplatin + *C. aurantium* (200 mg/kg)	261 ± 8.0^*∗∗*^	220 ± 7.9^*∗∗∗*^	241 ± 8.3^*∗∗∗*^	229 ± 11^*∗∗∗*^
Cisplatin + *C. aurantium* (400 mg/kg)	263 ± 8.2^*∗∗*^	229 ± 5.6^*∗∗∗*^	282 ± 8.5^*∗∗∗*^	270 ± 9.3^*∗∗∗*^

Statistical analysis was done through one-way analysis of variance (ANOVA) trailed by Bonferroni post hoc test for all groups in respective days. The results are considered significant (^*∗*^) if *p* < 0.005. ^*∗∗*^*p* < 0.05, ^*∗∗∗*^*p* < 0.05. Results were compared in a column with the respective control values.

**Table 2:** Effect of crude extract of *C. aurantium* on urine output in rats treated with cisplatin.

Days of observation				
Groups	0^th^ day	7^th^day	14^th^day	21^st^day
Control	7.2 ± 1.8	7.0 ± 1.8	7.5 ± 1.7	6.3 ± 1.1
Cisplatin	7.0 ± 1.8	31 ± 2.2^*∗∗∗*^	31 ± 3.0^*∗∗∗*^	33 ± 1.5^*∗∗∗*^
Cisplatin + *C. aurantium* (200 mg/kg)	8.0 ± 1.7^*∗∗*^	23 ± 1.8^*∗∗∗*^	24 ± 8.3^*∗∗∗*^	22 ± 1.2^*∗∗∗*^
Cisplatin + *C*. *aurantium* (400 mg/kg)	7.2 ± 1.6^*∗∗*^	15 ± 1.4^*∗∗∗*^	16 ± 1.6^*∗∗*^	17 ± 0.88^*∗∗∗*^

Statistical analysis was done through one-way analysis of variance (ANOVA) trailed by Bonferroni post hoc test for all groups in respective days. The results are considered significant (^*∗*^) if *p* < 0.005. ^*∗∗*^*p* < 0.05, ^*∗∗∗*^*p* < 0.05. Results were compared in a column with the respective control values.

**Table 3:** Effect of crude extract of *C. aurantium* on kidney weights.

Groups	Kidney weight
Normal control	0.41 ± 0.047
Cisplatin	1.7 ± 0.088^*∗∗∗*^
Cisplatin + *C. aurantium* (200 mg/kg)	1.1 ± 0.044
Cisplatin + *C. aurantium* (400 mg/kg)	0.72 ± 0.042

Statistical analysis was done through one-way analysis of variance (ANOVA) trailed by Bonferroni post hoc test for all groups in respective days. The results are considered significant (^*∗*^) if *p* < 0.005. ^*∗∗∗*^*p* < 0.05. Results were compared in a column with the respective control values.

**Table 4:** Effect of crude extract of *C. aurantium* on plasma sodium and potassium levels in rats treated with cisplatin.

		Days of observation			
Parameter	Groups	0^th^ day	7^th^ day	14^th^ day	21^st^ day
Plasma sodium (mEq/L)	Control	139 ± 6.0	179 ± 5.9	178 ± 4.2	139 ± 6.1
	Cisplatin	146 ± 4.9	94 ± 8.0^*∗∗∗*^	106 ± 6.3^*∗∗∗*^	103 ± 6.6^*∗∗∗*^
	Cisplatin + *C. aurantium* (200 mg/kg)	144 ± 4.8^*∗∗*^	123 ± 5.9^*∗∗∗*^	130 ± 4.9^*∗∗∗*^	122 ± 7.1^*∗∗∗*^
	Cisplatin + *C. aurantium* (400 mg/kg)	149 ± 4.2^*∗∗∗*^	150 ± 5.5^*∗∗∗*^	155 ± 5.7^*∗∗∗*^	144 ± 6.4
Plasma potassium (mEq/L)	Control	5.9 ± 0.34	5.5 ± 0.45	6.1 ± 0.33	5.9 ± 0.27
	Cisplatin	5.7 ± 0.17	1.6 ± 0.20^*∗∗∗*^	2.2 ± 0.27^*∗∗∗*^	2.2 ± 0.28^*∗∗∗*^
	Cisplatin + *C. aurantium* (200 mg/kg)	5.7 ± 0.42	3.1 ± 0.20^*∗∗∗*^	3.4 ± 0.24^*∗∗∗*^	3.6 ± 0.17^*∗∗∗*^
	Cisplatin + *C. aurantium* (400 mg/kg)	5.8 ± 0.23	4.3 ± 0.19^*∗∗∗*^	4.7 ± 0.29^*∗∗∗*^	4.7 ± 0.33^*∗∗∗*^

Statistical analysis was done through one-way analysis of variance (ANOVA) trailed by Bonferroni post hoc test for all groups in respective days. The results are considered significant (^*∗*^) if *p* < 0.005. ^*∗∗*^*p* < 0.05, ^*∗∗∗*^*p* < 0.05. Results were compared in a column with the respective control values.

**Table 5:** Effect of crude extract of *C*. *aurantium* on urinary sodium and potassium levels in rats treated with cisplatin.

		Days of observation			
Parameter	Groups	0^th^ day	7^th^ day	14^th^ day	21^st^ day
Urinary sodium (mEq/24 h)	Control	172 ± 4.0	180 ± 9.2	190 ± 7.3	170 ± 8.4
	Cisplatin	169 ± 4.5	300 ± 11^*∗∗∗*^	303 ± 11^*∗∗∗*^	347 ± 17^*∗∗∗*^
	Cisplatin + *C. aurantium* (200 mg/kg)	172 ± 8.0	261 ± 8.0^*∗∗∗*^	267 ± 7.8^*∗∗∗*^	296 ± 11^*∗∗∗*^
	Cisplatin + *C. aurantium* (400 mg/kg)	164 ± 6.2^*∗∗∗*^	218 ± 8.3^*∗∗∗*^	228 ± 8.7^*∗∗∗*^	247 ± 7.8^*∗∗∗*^
Urinary potassium (mEq/24 h)	Control	3.8 ± 0.35	3.0 ± 0.25	3.3 ± 0.35	3.5 ± 0.19
	Cisplatin	3.4 ± 0.36^*∗∗∗*^	4.3 ± 0.19^*∗∗∗*^	4.7 ± 0.29^*∗∗∗*^	5.1 ± 0.34^*∗∗∗*^
	Cisplatin + *C. aurantium* (200 mg/kg)	3.5 ± 0.26^*∗∗∗*^	3.1 ± 0.20	3.4 ± 0.24	3.6 ± 0.17
	Cisplatin + *C. aurantium* (400 mg/kg)	3.3 ± 0.51^*∗∗∗*^	1.6 ± 0.20^*∗∗∗*^	2.2 ± 0.27^*∗∗∗*^	2.8 ± 0.32^*∗∗∗*^

Statistical analysis was done through one-way analysis of variance (ANOVA) trailed by Bonferroni post hoc test for all groups in respective days. The results are considered significant (^*∗*^) if *p* < 0.005. ^*∗∗∗*^*p* < 0.05. Results were compared in a column with the respective control values.

**Table 6:** Effect of crude extract of *C. aurantium* on absolute creatinine clearance in rats treated with cisplatin.

		Days of observation			
Parameter	Groups	0^th^ day	7^th^ day	14^th^ day	21^st^ day
Absolute creatinine clearance	Control	0.27 ± 0.024	0.42 ± 0.016^#^	0.40 ± 0.011	0.86 ± 0.024
	Cisplatin	0.26 ± 0.036^*∗*^	0.33 ± 0.019^*∗∗∗*^	0.31 ± 0.014^*∗∗∗*^	0.40 ± 0.028^*∗∗*^
	Cisplatin + *C. aurantium* (200 mg/kg)	0.26 ± 0.028^*∗∗*^	0.49 ± 0.015^*∗∗∗*^	0.53 ± 0.037^*∗∗∗*^	0.66 ± 0.054
	Cisplatin + *C. aurantium* (400 mg/kg)	0.29 ± 0.037^*∗∗∗*^	0.61 ± 0.030^*∗∗∗*^	0.73 ± 0.031^*∗∗∗*^	1.2 ± 0.096^*∗*^

Statistical analysis was done through one-way analysis of variance (ANOVA) trailed by Bonferroni post hoc test for all groups in respective days. The results are considered significant (^*∗*^) if *p* < 0.005. ^*∗*^*p* < 0.05, ^*∗∗*^*p* < 0.05, ^*∗∗∗*^ indicates *p* < 0.05. Results were compared in a column with the respective control values.

**Table 7:** Effect of crude extract of *C. aurantium* on absolute potassium excretion in rats treated with cisplatin

		Days of observation			
Parameter	Groups	0 day	7^th^ day	14^th^ day	21^st^ day
Absolute excretion of potassium	Control	0.0062 ± 0.00070	0.0039 ± 0.00087	0.0029 ± 0.00027	0.0024 ± 0.00048
	Cisplatin	0.0055 ± 0.00089^*∗∗∗*^	0.0057 ± 0.00042^*∗∗∗*^	0.0061 ± 0.00037	0.0067 ± 0.00092^*∗∗∗*^
	Cisplatin + *C. aurantium* (200 mg/kg)	0.0055 ± 0.0096^*∗∗∗*^	0.0043 ± 0.00069^*∗∗∗*^	0.0041 ± 0.00063	0.042 ± 0.00055^*∗∗∗*^
	Cisplatin + *C. aurantium* (400 mg/kg)	0.0057 ± 0.0071^*∗∗∗*^	0.0023 ± 0.00045^*∗∗∗*^	0.0018 ± 0.00051	0.034 ± 0.00057^*∗∗∗*^

Statistical analysis was done through one-way analysis of variance (ANOVA) trailed by Bonferroni post hoc test for all groups in respective days. The results are considered significant (^*∗*^) if *p* < 0.005. ^*∗∗∗*^*p* < 0.05. Results were compared in a column with the respective control values.

**Table 8:** Effect of crude extract of *C. aurantium* on urinary sodium to potassium ratio in rats treated with cisplatin.

		Days of observation			
Parameter	Groups	0^th^ day	7^th^ day	14^th^ day	21^st^ day
Urinary Na^2+/^K^+^ ratio	Control	4.7 ± 0.56	3.4 ± 0.48	3.3 ± 0.45	3.7 ± 0.44^*∗∗∗*^
	Cisplatin	5.1 ± 0.47^*∗*^	16 ± 1.3^*∗∗∗*^	19 ± 0.83^*∗∗∗*^	20 ± 3.7^*∗∗∗*^
	Cisplatin + *C. aurantium* (200 mg/kg)	6.0 ± 0.68^*∗∗∗*^	11 ± 1.4^*∗∗∗*^	8.3 ± 0.67^*∗∗∗*^	10 ± 1.2^*∗∗∗*^
	Cisplatin + *C. aurantium* (400 mg/kg)	4.7 ± 0.42	5.7 ± 0.61^*∗∗∗*^	4.6 ± 0.43^*∗∗∗*^	6 ± 0.81^*∗∗∗*^

Statistical analysis was done through one-way analysis of variance (ANOVA) trailed by Bonferroni post hoc test for all groups in respective days. The results are considered significant (^*∗*^) if *p* < 0.005. ^*∗*^*p* < 0.05, ^*∗∗∗*^*p* < 0.05. Results were compared in a column with the respective control values.

**Table 9:** Effect of crude extract of *C. aurantium* on fractional excretion of potassium and sodium in rats treated with cisplatin.

		Days of observation			
Parameter	Groups	0^th^ day	7^th^ day	14^th^ day	21^st^ day
Fractional excretion of potassium	Control	3.8 ± 0.48	5.6 ± 1.4	10 ± 2.3	9.2 ± 1.6
	Cisplatin	3.8 ± 0.29	17 ± 3.2^*∗∗∗*^	24 ± 3.5^*∗∗∗*^	26 ± 2.8^*∗∗∗*^
	Cisplatin + *C. aurantium* (200 mg/kg)	3.6 ± 0.23^*∗∗∗*^	12 ± 2.0^*∗∗*^	19 ± 2.6^*∗∗∗*^	19 ± 2.1^*∗∗∗*^
	Cisplatin + *C. aurantium* (400 mg/kg)	3.7 ± 0.48^*∗∗∗*^	8.3 ± 1.8^*∗∗∗*^	14 ± 2.3^*∗∗∗*^	13 ± 1.6^*∗∗∗*^
Fractional excretion of sodium	Control	0.28 ± 0.036	0.30 ± 0.06	0.33 ± 0.09	0.30 ± 0.05
	Cisplatin	0.26 ± 0.044	0.77 ± 0.25^*∗∗∗*^	0.61 ± 00.16^*∗∗∗*^	0.69 ± 0.09^*∗∗∗*^
	Cisplatin + *C. aurantium* (200 mg/kg)	0.27 ± 0.07^*∗∗*^	0.62 ± 0.22^*∗∗∗*^	0.49 ± 0.11^*∗∗∗*^	0.53 ± 0.06^*∗∗∗*^
	Cisplatin *C. aurantium* (400 mg/kg)	0.32 ± 0.14^*∗∗*^	0.43 ± 0.12^*∗∗∗*^	0.03 ± 0.009^*∗∗∗*^	0.35 ± 0.05^*∗∗∗*^

Statistical analysis was done through one-way analysis of variance (ANOVA) trailed by Bonferroni post hoc test for all groups in respective days. The results are considered significant (^*∗*^) if *p* < 0.005. ^*∗∗*^*p* < 0.05, ^*∗∗∗*^*p* < 0.05. Results were compared in a column with the respective control values.

## Data Availability

The data used to support the findings of this study are available from the corresponding author upon request.
